# Cellular and Molecular Mechanisms of Heterotopic Ossification in Fibrodysplasia Ossificans Progressiva

**DOI:** 10.3390/biomedicines12040779

**Published:** 2024-04-02

**Authors:** Loreilys Mejias Rivera, Eileen M. Shore, Foteini Mourkioti

**Affiliations:** 1 Cell and Molecular Biology, Genetics and Epigenetics Graduate Program, University of Pennsylvania, Philadelphia, PA 19104, USA; loreilys.mejias@pennmedicine.upenn.edu; 2 Department of Orthopaedic Surgery, Perelman School of Medicine, University of Pennsylvania, 3450 Hamilton Walk, Philadelphia, PA 19104, USA; 3 Center for Research in FOP and Related Disorders, Perelman School of Medicine, University of Pennsylvania, Philadelphia, PA 19104, USA; 4 Department of Genetics, Perelman School of Medicine, University of Pennsylvania, Philadelphia, PA 19104, USA; 5 Department of Cell and Developmental Biology, Perelman School of Medicine, University of Pennsylvania, Philadelphia, PA 19104, USA; 6 Musculoskeletal Program, Penn Institute for Regenerative Medicine, Perelman School of Medicine, University of Pennsylvania, Philadelphia, PA 19104, USA

**Keywords:** FOP, heterotopic ossification, ACVR1 mutation, fibro-adipogenic progenitors, HO progenitor cells, muscle regeneration, musculoskeletal disease

## Abstract

Fibrodysplasia ossificans progressiva (FOP) is a debilitating genetic disorder characterized by recurrent episodes of heterotopic ossification (HO) formation in muscles, tendons, and ligaments. FOP is caused by a missense mutation in the *ACVR1* gene (activin A receptor type I), an important signaling receptor involved in endochondral ossification. The *ACVR1^R206^^H^* mutation induces increased downstream canonical SMAD-signaling and drives tissue-resident progenitor cells with osteogenic potential to participate in endochondral HO formation. In this article, we review aberrant ACVR1^R206H^ signaling and the cells that give rise to HO in FOP. FOP mouse models and lineage tracing analyses have been used to provide strong evidence for tissue-resident mesenchymal cells as cellular contributors to HO. We assess how the underlying mutation in FOP disrupts muscle-specific dynamics during homeostasis and repair, with a focus on muscle-resident mesenchymal cells known as fibro-adipogenic progenitors (FAPs). Accumulating research points to FAPs as a prominent HO progenitor population, with *ACVR1^R206^^H^* FAPs not only aberrantly differentiating into chondro-osteogenic lineages but creating a permissive environment for bone formation at the expense of muscle regeneration. We will further discuss the emerging role of *ACVR1^R206^^H^* FAPs in muscle regeneration and therapeutic targeting of these cells to reduce HO formation in FOP.

## 1. Introduction

Fibrodysplasia ossificans progressiva (FOP) is a rare but devastating autosomal dominant genetic disorder characterized by spontaneous or trauma-induced progressive extra-skeletal bone formation, called heterotopic ossification (HO), in skeletal muscles, tendons, and ligaments [1]. HO forms through endochondral ossification, the process that creates most bones during embryonic development [2]. Endochondral ossification occurs through the formation of a cartilaginous structure followed by cartilage resorption and its replacement by bone [2]. In the case of FOP, endochondral osteogenesis occurs extra-skeletally, replacing soft connective tissues with bone tissue [1]. Causative missense mutations for FOP occur in the *ACVR1* gene, which encodes a type I bone morphogenic protein (BMP) receptor [3], with *ACVR1^R206H^* being the most prominent mutation, occurring in an estimated 97% of FOP patients [4,5,6,7]. The prevalence of FOP has been estimated to be approximately 0.6–1.39 per million inhabitants, with around 900 confirmed cases worldwide [1,8,9,10]. No sex, racial, or ethnic patterns have been observed in FOP patients [1,8,9,11,12,13]; however, not enough populations have been investigated to conclusively exclude these confounding factors. Around half of FOP patients are misdiagnosed, most commonly with cancer, which has often lead to a 5–6-year delay in the correct diagnosis [11,12,13,14]. While misdiagnoses are common in many rare diseases, increasing FOP awareness within the medical community and globally is necessary to lessen preventable harm to patients due to intramuscular injections, invasive biopsies, and surgical procedures [14,15].

Most patients with FOP are born with congenital malformation of the great toe, but otherwise appear normal at birth [1]. Children with FOP start experiencing unpredictable episodes of painful soft tissue swelling, known as flare-ups, around the age of 5, but onset varies per individual [6,11,12,16]. Flare-ups often lead to irreversible HO along the body and joints; however, ectopic bone formation has also been reported in the absence of flare-ups [6,11,12,16]. Even though flare-ups and new episodes of HO can be triggered by muscular trauma, they also occur spontaneously, supporting the critical role of the immune system in the pathogenesis of FOP. However, the cause of these local inflammatory symptoms remains unclear. Heterotopic bone forms more commonly in the upper body before age 8, while over time, HO formation occurs more distally and in the lower limbs [11,12,16]. Cumulative HO formation restricts range of motion, causes severe pain, and gradually immobilizes patients, reducing their quality of life. Most heterotopic bone forms during adolescence and young adulthood; with age, accumulation of new HO volume decreases, which may be attributed to reduced HO initiation and tissue availability [11]. Disease progression leads to most patients needing aids, assistive devices, and adaptations (AADAs) by their 20s [11]. Progressive HO shortens the lifespan of individuals with FOP, with 56 years as the median life expectancy [15]. The leading cause of death among patients is thoracic insufficiency syndrome, caused by cardiorespiratory failure due to ossification of the rib cage [15].

There is no current cure for FOP patients, though, in a significant advancement, Palovarotene, a selective retinoic acid receptor gamma (RARy) agonist, has been recently approved by the FDA in the United States (US) and by respective agencies in Canada and Australia as a treatment to reduce new HO formation [17,18,19,20]. However, concerns for skeletal growth were raised during clinical trials and a pre-clinical study [18,20,21], resulting in Palovarotene only being approved for patients aged ≥8 and ≥10 years for females and males, respectively in the US, Australia, and Canada [17,20]. Considering these advancements, research into FOP pathogenesis and the development of other effective treatments, some already in clinical trials [22], are necessary so children with FOP can be treated, given that significant HO progression occurs during childhood. 

In the last few years, considerable progress in FOP research has led to the identification of cellular progenitor(s) of HO. Tissue-resident mesenchymal progenitors have been implicated as cellular origins of ectopic cartilage [23]. The development of treatments targeting these progenitors has potential to relieve the burden of HO in patients. In this review, we highlight the cellular sources of HO, focusing on the emerging role of fibro-adipogenic progenitors (FAPs) in the pathogenesis of FOP.

## 2. ACVR1-Mediated Signaling and FOP

ACVR1 is one of the cell surface receptors that mediate bone morphogenic protein (BMP) signaling [24,25,26]. BMP signaling has been extensively studied for its role in skeleton patterning, endochondral skeletal development, growth plate growth, and chondro-osteogenic differentiation [24]. *ACVR1^KO^* studies show the receptor’s crucial role in endochondral bone formation and craniofacial development, as well as non-bone processes such as cardiac development and neurogenesis [25]. BMPs also participate in a variety of biological processes such as cell proliferation, differentiation, embryogenesis, and development, as well as in adult tissue homeostasis in bone and other tissues [26]. ACVR1 signals through suppressor of mothers against decapentaplegic (SMAD)-dependent (canonical) and SMAD-independent (non-canonical) pathways [24,25,26]. This section will define the role of ACVR1 in bone regulation and homeostasis, including how dysregulated ACVR1^R206H^-receptor signaling drives FOP pathogenesis.

**Figure 1:**
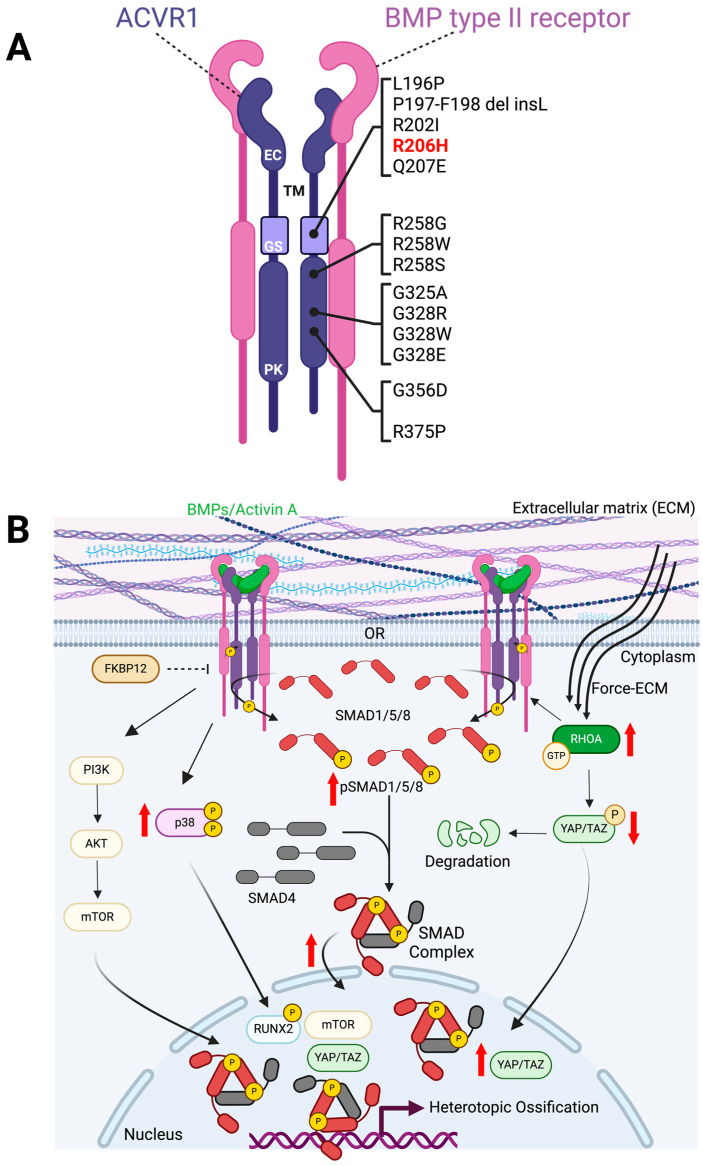
ACVR1 mutations and dysregulated downstream signaling in FOP. (**A**) Heterotetrameric receptor complex of BMP type II receptors (pink) and BMP type I receptors, ACVR1 (purple). ACVR1 has 4 domains, an extracellular (EC) binding domain, where ligands can bind and induce or inhibit downstream signaling, and a transmembrane (TM) domain. Known FOP causative mutations occur in the glycine–serine (GS) rich activation domain and the protein kinase (PK) domain. Both the GS and PK domains are important for downstream signaling activation. The most recurrent mutation found in FOP patients is R206H (red), located in the GS domain. (**B**) ACVR1^R206H^-mediated signaling in FOP. Signaling can be ligand-dependent (BMPs/Activin A) as depicted here or ligand-independent (not shown). Canonically, ACVR1^R206H^ hyperactivity induces increased SMAD1/5/8 phosphorylation and SMAD complex localization into the nucleus, ultimately increasing the expression of target osteogenic genes. Non-canonically, ACVR1^R206H^ induces PI3K-AKT-mTOR signaling; increases p38 phosphorylation and activity, later activating downstream transcription factor targets (i.e., RUNX2); and increases RhoA activation and mediated downstream effectors to increase YAP/TAZ nuclear localization to induce HO formation. Negative regulation by FKBP12 is affected and reduced in ACVR1^R206H^ by changes in binding affinity. Abbreviations: phosphoinositide 3-kinase (PI3K), protein kinase B (AKT), mammalian target of rapamycin (mTOR), runt-related transcription factor 2 (RUNX2), Ras homolog gene family member A (RhoA), yes-associated protein (YAP), tafazzin (TAZ), and 12-kDa FK506-binding protein (FKBP12). Created with BioRender.com.

Figure 1 summarizes how canonical and noncanonical ACVR1-mediated signaling is altered in FOP. Specific pharmacological targeting of these pathways has been studied to ablate HO formation, with multiple preclinical studies showing promise for new FOP treatments. 

### 2.1. Canonical Signaling

BMP pathway signal transduction is mediated through the canonical SMAD-dependent pathway [24]. BMP ligands (such as BMP-4/7 heterodimers) bind to a heterotetrameric complex composed of two type I and II BMP receptor heterodimers [26]. Upon ligand binding, the type II BMP receptor trans-phosphorylates and activates the type I receptor kinase to phosphorylate SMAD1/5/8 transcription factors, which in turn bind and form a complex with SMAD4, and subsequently translocate into the nucleus, bind to Smad-binding elements (SBE) and regulate target gene expression [26,27]. 

Numerous BMP-responsive genes have been identified, such as known osteogenic genes *Runx2*, *Dlx5*, and *Osx* [27,28]. In addition to targeting genes involved in osteoblast differentiation, BMP-SMAD signaling is essential for endochondral ossification, the process of forming bone through a cartilaginous intermediary. ACVR1, also known as ALK2, is required for proper chondrocyte proliferation and differentiation, as mice with cartilage-specific *Acvr1* knock-out (*Acvr1^CKO^*) presented craniofacial and axial defects as well as reduced pSMAD1/5 activity [29]. Chondrocyte gene expression is majorly mediated through SMAD-dependent pathways, since murine models of conditional *Smad1* chondrocyte-specific deletion exhibit growth plate shortening and delayed calvarial bone development, while *Smad5^CKO^* exhibited severe chondrodysplasia [27,30,31]. BMP signaling regulates chondrocyte proliferation and differentiation by maintaining SRY-box transcription factor 9 (*SOX9)* expression, a master chondrogenic transcription factor, and inducing transcription and activation of RUNX2, which is required for chondrocyte hypertrophy [32,33]. SMADs interact with multiple downstream transcription factors, co-activators, and chromatin remodelers to further induce target gene expression [27]. However, the SMAD target transcriptome has been shown to vary by cell type and be context-dependent. Lineage-specific transcription factors, the chromatin accessibility landscape, and other DNA-binding proteins define the binding patterns and transcriptional activity of pSMAD1/5/8 in a cell type-specific manner [27,34,35]. 

### 2.2. Non-Canonical Signaling

ACVR1 signaling via SMAD-independent pathways also plays critical roles in skeletal development and osteogenic differentiation through mitogen-activated protein kinases (MPAKs) [24,36]. BMPs also induce receptor-mediated phosphorylation/activation of MAPKs [36]. The TAK1–MKK–MAPK pathway leads to p38MAPK, ERK, and/or JNK activation [36]. TAK1, a MAPK kinase kinase (MAPKKK) family member, is essential for joint and cartilage development [37]. Cartilage-specific Tak1-deficient mice display growth plate and articular cartilage defects, with reduced chondrogenic genes and *Sox9/5/6* [37]. Following TAK1 activation, MAPK (including p38) phosphorylate master osteogenic transcription factors *RUNX2*, *DXL-5*, and *OSX* [36,37]. Phosphorylation promotes transcription factor activity and facilitates binding to co-factors [38]. MAPKs also positively regulate *RUNX2* and *OSX* expression [24]. Reciprocal activation occurs in both canonical and non-canonical BMP pathways. RUNX2 depends on MAPK phosphorylation to enable complex formation with the SMAD complex to further induce transcription of downstream osteogenic targets [39]. Acvr1 also signals through the Pi3k/Akt/mTOR pathway, and Pi3k has been shown to mediate Smad1 stabilization via glycogen synthase kinase 3 (Gsk3) activation, supporting osteogenesis [40]. 

Accumulating evidence has linked BMP signaling to mechanosensitivity, the ability of a cell to interpret its microenvironment via mechanical cues including force and stress as well as substrate stiffness and topology [41]. BMPs activate members of the Rho-like GTPases family, like RhoA, which activate the downstream master mechano-transduce transcription factors Yap/Taz to regulate cytoskeletal dynamics [41]. BMPs additionally activate members of the Rho-like GTPases to regulate cytoskeletal dynamics, including contractile tension and motility, and osteogenic differentiation [36,42]. Rho A-mediated mechanotransduction also facilitates Smad1/5/8 activation, linking not only canonical and non-canonical Acvr1 signaling but also BMP and mechanosignaling [41,42].

### 2.3. ACVR1^R206H^ Mutation and Altered Downstream Signaling

The R206H mutation is located within the glycine–serine (GS) domain of *ACVR1* [3]. All known causal FOP mutations occur in either the *ACVR1* gene GS domain or the protein kinase domain, both intracellular regions critical for downstream signal transduction (Figure 1A) [3,4,5,6,7,25]. ACVR1 receptor forms heterodimers with another type I receptor, which then forms a complex with type II BMP receptors to induce downstream signaling. However, it was recently shown that ACVR1^R206H^ does not require a type I receptor heterodimer partner for signaling [43,44]. This means ACVR1^R206H^ is not constrained by the same receptor partner requirements and regulatory mechanisms as ACVR1. These findings led researchers to theorize a capacity of ACVR1^R206H^ to signal in response to new ligands. It also implies an acquired ability to signal in new combinations of receptor complexes, although more studies are needed to confirm this. Overall, variations in signaling output might be due to the numerous potential ligand–receptor and receptor–receptor pairings in transforming growth factor-beta (TGFβ)/BMP signaling, which may differ from cell to cell or within the same cell in various contexts, and which are further perturbed by mutations in *ACVR1* [45]. 

In addition to altered type I receptor-mediated regulation of BMP receptor complex signaling, ligand regulation of signaling by ACVR1^R206H^ in FOP is also perturbed, with the mutant receptors conferring both ligand-independent activity and ligand-dependent hyperactivity (Figure 1B). ACVR1^R206H^ shows mild ligand-independent activation and hypersensitivity to BMP ligands in FOP patient-derived cells, cell lines, and zebrafish models [44,46,47,48,49]. Canonically, the mutant receptor increases SMAD1/5/8 phosphorylation and nuclear localization and upregulates target gene expression (Figure 1B) [46,47]. However, further studies are needed to determine the mechanisms SMADs employ to induce endochondral differentiation in ACVR1^R206H^ cells. 

ACVR1^R206H^ hypersensitivity to BMP ligands has been well documented [46,47]. Recently, activin A was shown to induce SMAD1/5/8 activation via ACVR1^R206H^ in vitro via aberrant ligand activation of BMP signaling [50]. Activin A, a member of the TGFβ/BMP family of ligands, predominantly signals through the type I receptor ACVR1B and type II receptor ACVR2A/B, leading to downstream activation of the SMAD2/3 pathway [51,52]. Activin A has been shown to function as a competitive antagonist, binding to ACVR1 and type II BMP receptor ACVR2A/B and inhibiting SMAD1/5/8 phosphorylation [43,51,52]. ACVR1^R206H^ neofunction to activin A (meaning the mutation confers novel receptor activity in response to activin A) has been investigated both in vitro and in vivo. In a conditional Acvr1^R206H^ knock-in mouse model, HO was induced by activin A injection and abrogated by antibodies against activin A, indicating that this ligand is sufficient to drive HO in FOP [43]. In comparison, recombinant BMP ligands implanted into in vivo sites are also sufficient to drive HO formation, even in the absence of the FOP mutation [53,54].

In a more recent model, activin A treatment was shown to promote osteogenic differentiation of HO progenitor cells in vitro and drive ectopic ossification when the *Acvr1* mutation is solely expressed in Tie2-lineage mouse cells [55]. Although evidence supports activin A’s role in inducing heterotopic ossification, data from previous studies have shown activin A induction of SMAD1/5/8 activation in the context of ACVR1^R206H^ varies per cell type [56,57]. Activin A has been shown to activate SMAD1/5/8 via ACVR1 in myeloma cell lines in the absence of *ACVR1* mutation [57]. However, FOP patient-derived endothelial cells from induced pluripotent human stem cells did not show SMAD1/5/8 activation after activin A treatment, and no significant differences have been found in the serum levels of activin A, BMP4, or BMP6 in FOP patients [56,58]. Altogether, these data suggest that activin A signaling activity may be variable, further highlighting the need to investigate the cellular context needed for activin A to induce or inhibit downstream ACVR1-mediated signaling. 

The *ACVR1* R206H mutation has also been shown to alter mutant receptor binding with regulators that are important to inhibit BMP signaling in the absence of receptor activation through ligand binding (Figure 1B) [59,60,61]. In vitro, the mutant ACVR1 receptor shows reduced binding to FKBP12, a negative regulator of type I receptors, implying that increased BMP pathway signaling in cells may be attributable to decreased binding of this inhibitory factor [59,60,61].

Mutant ACVR1 also dysregulates non-canonical pathways (Figure 1B) [46,62,63,64,65,66]. The FOP mutation has been shown to increase MAPK phosphorylation [66]. FOP lymphocytes presented increased levels of p38 phosphorylation and p38 MAPK activity when treated with BMPs [66]. Acvr1^R206H^ has been shown to induce PI3ka and mTOR signaling for HO formation, whereas mTOR complex and PI3k inhibitors have been shown to reduce HO in FOP mice [62,63,64,65].

Acvr1^R206H^ cells actively misinterpret their microenvironment as stiffer, further enhancing their misdifferentiation [67,68]. Acvr1^R206H^ cells display altered sensitivity to mechanical stimuli, sensing soft microenvironments as stiff, increasing RhoA activation and downstream effectors (pCofilin, pMLC2 (myosin light chain 2), YAP/TAZ) and osteogenic differentiation [67,68]. In an FOP mouse model with an engineered constitutively active mutant of *Acvr1* (*Acvr1^Q207D^*; not found in FOP patients), Yap was also shown to be upregulated and a key player for HO formation, as its genetic deletion ablated extraskeletal bone formation [69].

In summary, although much has been discovered over the years regarding the dysregulation of ACVR1^R2206H^ signaling pathways (canonical and non-canonical), more research is needed to determine how different ACVR1^R206H^ cell types with diverse receptor expression profiles and downstream signaling pathways respond to ligands and whether additional molecules induce ligand-dependent signaling in a context dependent manner.

## 3. Cellular Progenitors of Heterotopic Ossification in FOP

Bone marrow progenitors and hematopoietic cells were initially proposed to be sources of ectopic cartilage and bone in FOP, since hematopoietic mononuclear cells were observed in early pre-osseous lesions of patients [70]. However, bone marrow transplants in mice and an FOP patient proved ineffective, as there was no reduction of HO [71]. Lineage tracing studies have been used to test if endothelial, bone marrow, pericyte, smooth muscle, tendon progenitors, muscle stem, or muscle-resident mesenchymal cells contribute to endochondral HO in FOP *Acvr1^R206H^* or *Acvr1^Q207D^* mouse models (Table 1) [55,72,73,74]. These studies have been valuable to exclude the contribution of bone marrow, pericyte, smooth muscle, and muscle stem cells as major sources of HO progenitor cells in FOP [55,72,73,74]. Here, we describe the use of knock-in *Acvr1^R206H^* mice under the control of several Cre drivers, nongenetic mouse models of HO, and/or BMP-induced HO models to summarize the findings related to the cellular etiology of heterotopic bone in FOP.

### 3.1. Endothelial Cells

Tie2-lineage cells have been previously recognized as endothelial precursors in multiple stages of heterotopic ossification: fibroproliferation, chondrogenic, and osteogenic [53]. Utilizing a Cre-Lox system, researchers showed Tie2+ cells participated in HO development in both genetic and BMP-induced mouse models [74]. Upon induction of R206H expression by injection of *Adeno-Cre*, chondrogenic and osteogenic lesions were enriched in Tie2+ chondrocytes and osteoblasts, suggesting an endothelial origin of heterotopic cartilage and bone [74]. Examination of HO formation in a knock-in mouse model for FOP (*Acvr1^R206H/+^*) demonstrated abundant Tie2+ cells in all stages of extra-skeletal bone [76]. Although Tie2+ lineage cells were identified in heterotopic lesions, these progenitors were ultimately determined not to be of endothelial lineage [54]. Tie2, while predominantly labeling the endothelium in muscle, is also expressed in non-endothelial cells [77]. In a murine model with a GFP reporter driven by *Tie2-Cre* and another endothelial marker, CD31, transplantation of Tie2-GFP+CD31+ cells did not result in HO formation, but Tie2-GFP+CD31- cells did activate a bone-forming program in a BMP-2-induced HO model [54]. Further studies demonstrated that the Tie2+Pdgfrα+Sca1+ multipotent mesenchymal progenitor population, resident to the skeletal muscle interstitium, has the potential to differentiate into osteogenic, fibrogenic, or adipogenic lineages [54,78,79,80].

BMP2-induced HO in both nongenetic and genetic *Acvr1^R206H^* mouse models utilizing GFP-lineage labeling under *VE-Cadherin-Cre*, a classic endothelial marker important for cellular function [81], has shown that VE-Cadherin-lineage cells do not contribute to ectopic bone formation [54,55,72]. Although the subject still requires further investigation, these data suggest that HO progenitor cells are not likely to originate from endothelial cells. In a traumatic HO mouse model, vascular endothelial growth factor A (VEGFA) genetic deletion in Prrx1-lineage cells resulted in less HO volume formation [82]. Interestingly, it was *Prrx1*-expressing mesenchymal cells that mostly expressed *Vegfa* in HO lesions, instead of endothelial cells [82]. Whether or not endothelial cells contribute as HO progenitor cells, heterotopic bone formation requires a pro-angiogenic environment and participation of endothelial cells in HO formation [82,83]. In light of all of these advancements, it is important to not discount endothelial involvement in HO formation; although these may not be HO cells of origin, endothelial cells may still present another axis for developing novel therapeutic treatments. The section below contains more details on the contributions of mesenchymal cells to HO in FOP.

### 3.2. Muscle-Resident Mesenchymal Cells

In the course of investigating cell-type sources of ectopic bone, local mesenchymal stem cells emerged as a logical candidate for HO progenitors. Interestingly, muscle interstitium-resident-Pdgfrα+ mesenchymal progenitors had been previously identified by two independent research groups [54,79,80]. These progenitor cells were named fibro-adipogenic progenitors (FAPs) as they could differentiate into fibroblasts and adipocytes [54,79,80]. Under usual circumstances, FAPs proliferate upon muscle damage and enhance myogenic differentiation of muscle stem cells (MuSCs), and myofibers inhibit FAP adipogenesis [78,80], suggesting crosstalk between FAPs and MuSCs. FAP-depleted animal models display muscle atrophy under homeostatic circumstances, indicating that FAPs are essential for skeletal muscle regeneration and maintenance [84,85]. These findings strongly support a key role of FAPs in muscle repair and indicate that efficient regeneration requires the coordinated action of FAPs and MuSCs. In a key advancement for FOP research, it was demonstrated that Acvr1^R206H/+^ FAPs undergo aberrant endochondral ossification and give rise to spontaneous and injury-induced HO [55]. The mutation was expressed under either *Tie2-Cre* or *Pdgfrα-Cre*, with the latter exhibiting a more severe HO phenotype and earlier onset [55]. In another significant advancement, mutant FAPs were shown to not only contribute to HO in FOP, but also to actively miscommunicate with MuSCs to impair muscle regeneration [73]. (See Section 4.2 for more information on FOP muscle regeneration.)

In additional studies, an interstitial Mx1+ population found in muscles also was found to facilitate intramuscular, injury-dependent HO in an *Acvr1^R206H^* mouse model [72]. Although the Mx1-lineage cells were not identified as FAPs, they express Pdgfrα and are located in the muscle interstitium, signifying that they may be a subset of FAP cells [72]. In vitro experiments further showed that isolated Mx1+Sca1+Pdgfrα+ FOP-interstitial cells had significantly more osteogenic capacity compared to controls [72]. Altogether, these data support the conclusion that a muscle interstitial Mx1+ population, independently of the bone marrow-derived Mx1+ population, and potentially a subset of Pdgfra+ FAPs, have the potential to give rise to HO in FOP.

### 3.3. Tenocyte Progenitor Cells

Researchers hypothesized that scleraxis-positive (Scx+) tendon-derived stem cells and their equivalent populations in ligaments and fascia might contribute to tendon and ligament HO [72]. Spontaneous HO was evident in *Scx-Cre*; Acvr1^R206H^ mice in the tibialis anterior and patellar ligaments, the Achilles tendon, and the knee joints by 8–18 weeks of age [72]. Similarly, *Scx-Cre; Acvr1^Q207D^* mice had significant HO in the tendons, ligaments, and joints, albeit with increased severity and earlier onset [72]. In both models, ligaments and tendons were replaced with a chondrogenic matrix, with Scx+ lineage cells giving rise to most hypertrophic chondrocytes in HO lesions. Subsequently, another research group provided evidence that Scx+ lineage cells contribute to ectopic bone formation in skeletal muscle tissue by demonstrating that direct muscle damage with cardiotoxin (CTX) in Acvr1^Q207D^ mice induces muscle ossification [75]. These findings suggest a post-natal Scx+ subpopulation of connective tissue cells can undergo osteochondrogenesis following local damage or elevated ACVR1 signaling. In non-genetic traumatic HO mouse models of burn–tendon injury using Scx- or Pdgfra-lineage fluorescent reporters, researchers also showed that Tppp3+ tendon sheath progenitors cells give rise to HO [86]. These studies ultimately support the contributions of tenocyte progenitor cells in FOP and acquired HO.

Accumulating research points toward FAPs as predominant precursors of HO in nongenetic and genetic models [23,54,55,73,79,87,88]. Evidence supports the conclusion that HO progenitor cells in FOP are musculoskeletal tissue-resident mesenchymal cells with chondro-osteogenic potential (FAPs, tendon progenitor cells), which upon expression of activating ACVR1 mutations (such as R206H) are re-directed to form ectopic bone formation. It is also possible neighboring cells are recruited to participate in endochondral osteogenesis and contribute to heterotopic cartilage and bone formation along with Pdgfra+ progenitors, although such cells might not be the cellular initiators. In summary, tendon stem cells and their contribution to HO formation, in addition to muscle-resident FAP cells, is an exciting relatively new area of investigation. Clearly, more research is needed to further understand how mutant Scx+ progenitor cells may affect tendon tissue homeostasis and repair and HO.

## 4. Rising Role of Fibro-Adipogenic Progenitors in FOP

Since their initial description in 2010, FAPs have been widely studied for their prominent role in skeletal muscle homeostasis, regeneration, and disease [78,79,85,89,90,91,92]. FAPs are heterogeneous mesenchymal stromal cells that reside within the muscle interstitium [79,89,90]. They are key effectors of ECM deposition and scarring in adult muscle connective tissue and have important roles in guiding muscle regeneration via cell–cell signaling [79,89,93]. These multilineage progenitors can differentiate into fibrogenic, adipogenic, chondrogenic, and osteogenic lineages in vitro, while FAPs are also shown to aberrantly differentiate in disease, contributing to fibrosis, intramuscular fat deposition, and heterotopic ossification in vivo [23,54,55,73,79,88,90,94]. The recent recognition of FAPs as a significant source of pathologic ectopic bone formation in FOP animal models [55,73] brings forward the need to understand the mechanisms underlying the pathologic differentiation of FAPs in the presence of the *ACVR1* R206H mutation. In this section, we will discuss FAPs’ role across muscle regeneration (and in FOP), their heterogeneity, and their multifaceted contribution to heterotopic ossification, as well as discuss the implications of FAPs as a therapeutic target for FOP treatment.

### 4.1. New Insights into FOP Muscle Regeneration

Muscle-resident mesenchymal progenitors are characterized by Pdgfrα+, Sca1+, and CD34+ expression and are required for steady-state skeletal muscle maintenance [78,79,85,89,90]. Upon injury, FAPs exit quiescence, proliferate, and peak by 3–4 days post-injury (dpi), while by 5 dpi, they are cleared by macrophage-derived TNF-α-mediated apoptosis [79,89,95]. Throughout muscle repair (3–21 dpi), FAPs influence the microenvironment by secreting an intricate combination of extracellular matrix components, ligands, cytokines, and immune-modulatory molecules to communicate with MuSCs and immune cells and rebuild the matrix network in the muscle tissue [79,93]. FAP cytokine secretions are also important and necessary for efficient muscle regeneration. These cells secrete interleukin 33 (IL-33), Cxcl1, Cxcl5, and IL-10 to signal for Treg cell regulation, monocyte infiltration, and influence the ‘switch’ from a pro- to anti-inflammatory macrophage phenotype upon injury [93,96,97,98]. FAP-derived soluble molecules, such as follistatin, IL-6, and WNT1-inducible-signaling pathway protein, stimulate MuSC expansion and differentiation [78,99,100]. According to growing data, FAPs differentiate into fibroblasts and are the major biological source of regenerative extracellular matrix (ECM) deposition for muscle regeneration secreting collagens, laminins, and fibrillins [79,89,93]. By 21 dpi, muscle tissue function and morphology has been restored in healthy muscles [89].

**Figure 2:**
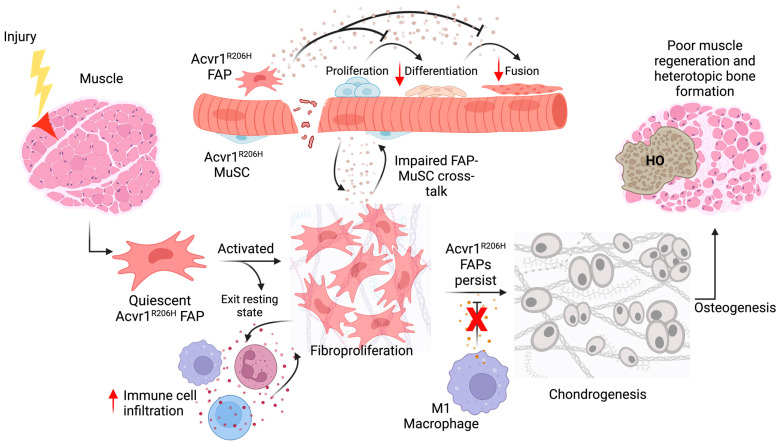
Model for FOP muscle regeneration. After muscle injury, quiescent ACVR1^R206H^ FAPs become activated and proliferate. However, abnormal FAP-derived soluble secretions decrease ACVR1^R206H^ MuSC myogenic commitment and ability to fuse to pre-existing myofibers. At the same time, there is increased immune cell infiltration, while FAPs resist macrophage-derived TNFa-mediated apoptosis and continue to accumulate within the FOP tissue, giving rise to aberrant osteochondrogenesis. This leads to reduced muscle regeneration and increased heterotopic ossification in FOP tissue. Created with BioRender.com.

Recently, the dynamics of muscle regeneration in FOP have been studied [73]. Utilizing a globally induced knock-in *Acvr1^R206H^* FOP mouse model, muscle repair in FOP and FAP behavior after muscle injury was evaluated [73]. *Acvr1^R206H^* FAPs had similar proliferation capacity compared to controls in vivo at 3, 5, and 7 dpi [73]. However, *Acvr1^R206H^* FAPs failed to be cleared by 5 dpi, as indicated by the decreased levels of apoptosis and the increased number of FAPs compared to controls [73]. Interestingly, muscle tissue had smaller regenerating myofibers, which indicates an impairment in muscle repair [73]. Although no differences in *Acvr1^R206H^* MuSC proliferation were seen in vivo, mutant MuSCs fail to form properly fused myofibers and exhibited impaired myogenic differentiation in vitro [73]. Remarkably, when the *Acvr1* mutation was expressed specifically in MuSC using a *Pax7-cre* line, the inefficient muscle regeneration seen in the globally induced model was not recapitulated, suggesting the effects on impaired muscle regeneration may be driven by the local tissue microenvironment and interactions with other mutant muscle-resident cells [73]. Indeed, co-culture of *Acvr1^R206H^* MuSCs with *Acvr1^R206H^* FAPs or conditioned media (CM) resulted in reduced or no fusion of myotubes, while mutant MuSCs co-cultured with control FAPs or CM were rescued from impaired differentiation [73]. These findings suggest that FAPs play an important role in influencing the myogenic activity of Acvr1^R206H^ MuSCs post-injury and that abnormal MuSC-FAP secretory profiles may lead to a previously unknown delay and inefficiency of skeletal muscle regeneration following injury. A proposed model for Acvr1^R206H^ FAP contribution to HO formation in FOP muscle is summarized in Figure 2.

### 4.2. Heterogeneity

Of note, recent single-cell transcriptomics has revealed Pdgfrα+, Sca1+, CD34+ FAPs are a heterogeneous population in both humans and mice [79,89]. Accumulating studies have described two FAP subpopulations present in mouse muscle: (i) FAP1, marked by Cxcl14+ and Lum+, which are enriched in ECM genes, and (ii) FAP2, which are Dpp4+ multipotent progenitors that express migratory and cell signaling genes [89,101,102,103,104]. However, increased FAP heterogeneity has been shown to take place in diseases and following injury [94]. In agreement, three FAP populations have been reported in humans: LUM+ FAP, FBN1+ FAP, and MME+ FAP subpopulations, which express high levels of collagens, fibrillins, and pro-adipogenic genes, respectively [105,106,107]. Still, much remains unknown regarding the effects of ACVR1^R206H^ on FAP biology, including their identity and the number of potential subpopulations, as well as information about their specific function, secretions, and transcriptome. Functional experiments are necessary to establish the mechanism in which ACVR1^R206H^ ‘reprograms’ FAPs to commit to osteochondrogenesis.

### 4.3. Contribution to Heterotopic Ossification

Accumulating evidence points toward FAPs, a multipotent muscle-resident mesenchymal cell population, as one of the sources of ectopic bone formation in muscle in both genetic and nongenetic forms of HO [23,54,55,73,79,88]. PDGFRα+ FAPs from both humans and mice readily undergo osteogenic differentiation when cultured in osteogenic conditions in vitro and contribute to HO in vivo via endochondral ossification in both BMP2-Induced and FOP mouse models [54,55,108]. Given the developments mentioned above, there is now a growing understanding of how HO progresses in FOP muscle. Upon CTX-skeletal muscle injury, Acvr1^R206H^ FAPs become activated, fibroproliferate (4 dpi), and accumulate by 5 dpi, contributing to the reported tissue stiffness (Figure 2) [67,73]. In parallel, there is increased and persistent immune cell infiltration from 2–6 dpi [109]. Mutant FAPs undergo chondrogenic differentiation by 7 dpi, and mature heterotopic bone is formed by 14 dpi (Figure 2) [55].

### 4.4. FAPs as a Therapeutic Target in FOP

Given the identification of FAPs as a cell-of-origin for HO in FOP, they have emerged as an important cell population to target therapeutically, including studies testing FAP-response to antibodies and drugs targeted to reduce their aberrant chondro-osteogenic differentiation.

Acvr1^R206H^ FAPs also spontaneously (in the absence of direct muscle injury) form HO in FOP mouse models, as well as undergo chondrogenic and osteogenic differentiation in vitro when cultured in basal media without the addition of exogenous ligands, which can be inhibited by anti-activin A antibodies [55,110]. Activin A has also been found to induce osteogenic differentiation of Acvr1^R206H^ FAPs in vitro [21], and when activin A in methylcellulose was intramuscularly injected into *Acvr1^R206H^;Tie2-Cre* mice, these also developed HO [55]. These developments have made activin A a target for human clinical trials to prevent HO formation. Garetosmab, an inhibitor of activin A, has been shown to reduce HO and flare-ups in FOP adults in phase II trials [111].

Palovarotene, a RARy agonist, is known to promote Smad1/5/8 protein degradation and downregulate BMP signaling in prechondrogenic cells and inhibit chondrogenesis and endochondral ossification [112]. Pre-clinical studies demonstrated the potential for Palovarotene as a potent HO-inhibitory drug, blocking both non-injury and injury-induced HO in FOP mouse models [21,113,114]. Indeed, Palovarotene inhibited osteogenic and chondrogenic differentiation of Acvr1^R206H^ FAPs in vitro and reduced HO in vivo in juvenile *Acvr1^R206H^;Pdgfrα-Cre* mice [21].

Both Palovarotene and activin A antibodies appear to affect FAPs/HO progenitor cells, however mechanisms of action are not completely understood. While Palovarotene remains the only FDA-approved drug in the United States for FOP, it is only approved for FOP patients >8 years for females, and >10 years for males due to concerns of negative effects on skeletal growth by inhibiting endochondral ossification [18]. Since young children with FOP have a high incidence of flare-ups and significant HO progression in early life [11], more research into FAPs and their role in FOP pathogenesis is needed to identify novel and specifically targeted therapies that can be used to effectively treat all FOP patients.

In summary, increasing evidence shows Acvr1^R206H^ FAPs are a source of HO in FOP, making these cells an ideal target to reduce HO formation in patients and maintain muscle and connective tissue health. Mutant FAPs not only aberrantly differentiate into chondrogenic and osteogenic lineages but actively suppress myogenic differentiation via secretions to impair muscle regeneration, ultimately replacing muscle with bone tissue. Still, much remains unknown regarding how Acvr1^R206H^ FAPs regulate muscle repair in FOP, as well as their paracrine interactions with immune cells known to be vital for FOP pathogenesis [109,115]. Future studies examining FAPs using multi-omics approaches have high potential to not only investigate the pathological differentiation of FAPs and associated gene expression, but also to elucidate the FAP secretome and spatial positional context in order to fully to understand the FAP-orchestrated changes in the muscle regeneration program.

## 5. Conclusions

Muscle-resident mesenchymal progenitors have been recognized as a main progenitor cell source of heterotopic ossification in genetic and non-genetic HO mouse models. However, other tissue-resident mesenchymal cells populations and subpopulations also appear to have relevance to HO formation and HO-forming potential, for example in Acvr1^R206H^ mouse models that have shown Scx+ tenocytes and FAPs can participate in extraskeletal bone formation. Further research is needed to understand the role each cell population plays, and to what extent these various cells contribute to endochondral HO within an FOP context. Additional important questions remain regarding the influence of ACVR1^R206H^ in FAP-guided muscle regeneration, heterogeneity, fate determination, gene expression, and cell–cell signaling. As we elucidate the mechanisms regulating cell differentiation toward heterotopic ossification, more effective and innovative therapies to prevent HO will be identified.

## Figures and Tables

**Table 1:** Transgenic FOP mouse models used to study/determine HO progenitor cells.

Transgenic Strain	Lineages Targeted	FOP Mouse Model Used	Formed HO?	In Vitro Phenotype	Reference(s)
*Vav1-Cre*	Bone marrow hematopoietic, Endothelial	*Acvr1^Q207D^*	No	-	[72]
*Cadh5-* *CreERT2* *Cadh5-Cre*	Mature endothelial	*Acvr1^Q207D^* *Acvr1^R206H^*	No	-	[55,72]
*sm22a-Cre*	Vascular smooth muscle; pericytes	*Acvr1^Q207D^*	No	-	[72]
*Cspg4-* *CreERT2*	NG2-expressing pericytes	*Acvr1^Q207D^*	No	-	[72]
*Pax7-Cre* *Pax7-CreERT2*	Muscle stem cell (MuSC)	*Acvr1^Q207D^* *Acvr1^R206H^*	No	*Acvr1^R206H^* MuSCs failed to form fused myofibers	[72,73]
*MyoD-iCre*	Myoblast	*Acvr1^R206H^*	No		[55]
*Myf6-Cre*	Myofiber	*Acvr1^Q207D^*	No		[72]
*Tie2-Cre*	Fibro-adipogenic progenitors (FAPs), endothelial	*Acvr1^R206H^*	Yes	*Acvr1^R206H^* FAPs show increased chondro-osteogenic differentiation and defective paracrine communication with MuSCs	[55]
*Pdgfrα-Cre*	FAPs, mesenchymal progenitors	*Acvr1^R206H^*	Yes		[55]
*Mx1-Cre*	Bone marrow, FAPs	*Acvr1^Q207D^* *Acvr1^R206H^*	Yes		[72]
*Scx-Cre* *Scx-CreERT2*	Tendon progenitors	*Acvr1^Q207D^* *Acvr1^R206H^*	Yes	*Acvr1^R206H^* tendon peogenitors show increased osteogenic differentiation when stimulated with ligands	[72,75]
